# Chemo‐Selective Single‐Cell Metabolomics Reveals the Spatiotemporal Behavior of Exogenous Pollutants During Xenopus Laevis Embryogenesis

**DOI:** 10.1002/advs.202305401

**Published:** 2023-12-20

**Authors:** Pengfei Li, Song Gao, Wanting Qu, Ying Li, Zhen Liu

**Affiliations:** ^1^ State Key Laboratory of Analytical Chemistry for Life Science School of Chemistry and Chemical Engineering Nanjing University Nanjing Jiangsu 210023 China

**Keywords:** boronate affinity extraction‐mass spectrometry, embryogenesis, exogenous pollutants, single cell analysis, targeted metabolomics

## Abstract

In‐depth profiling of embryogenesis‐associated endogenous and exogenous metabolic changes can reveal potential bio‐effects resulting from human‐made chemicals and underlying mechanisms. Due to the lack of potent tools for monitoring spatiotemporal distribution and bio‐transformation behavior of dynamic metabolites at single‐cell resolution, however, how and to what extent environmental chemicals may influence or interfere embryogenesis largely remain unclear. Herein, a zero‐sample‐loss micro‐biopsy‐based mass spectrometric platform is presented for quantitative, chemo‐selective, high‐coverage, and minimal‐destructive profiling of development‐associated cis‐diol metabolites, which are critical for signal transduction and epigenome regulation, at both cellular level and tissue level of Xenopus laevis. Using this platform, three extraordinary findings that are otherwise hard to achieve are revealed: 1) there are characteristically different cis‐diol metabolic signatures among oocytes, anterior and posterior part of tailbud‐stage embryos; 2) halogenated cis‐diols heavily accumulate at the posterior part of tailbud‐stage embryos of Xenopus laevis; 3) dimethachlon, a kind of exogenous fungicide that is widely used as pesticide, may be bio‐transformed and accumulated in vertebrate animals in environment. Thus, this study opens a new avenue to simultaneously monitoring intercellular and intraembryonic heterogeneity of endogenous and exogenous metabolites, providing new insights into metabolic remolding during embryogenesis and putting a warning on potential environmental risk.

## Graphical abstract

A micro‐biopsy‐based mass spectrometric platform is developed for in‐depth and minimal‐destructive characterization of cis‐diol metabolites during development from cellular level (single oocytes) to tissue level (different parts of tailbud‐stage embryos). The results reveal, for the first time, not only the multiscale heterogeneity of cis‐diols during embryogenesis but also a posterior‐preferred accumulation of halogenated cis‐diols in embryos.

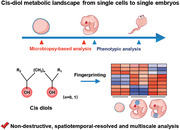

## 1 Introduction

Pesticides are widely used chemicals for food production but are also ubiquitous environmental pollutants.^[^ ^1^ ^]^ Typically, they were leveraged as agonists to disrupt metabolic pathways in invertebrate pests,^[^ ^2^ ^]^ but emerging evidence has shown that they may also act as exogenous disruptors to interrupt endocrine system of humans;^[^ ^3^ ^]^ and unfortunately, the influence may be magnified by long‐term bio‐accumulation through the food chain.^[^ ^4^ ^]^ What's more, by some estimates, overall 64 % of global agricultural land is at risk of pesticide pollution from more than one active ingredient now,^[^ ^1^ ^]^ resulting in huge potential risks to metabolic homeostasis of humans. Therefore, in‐depth characterization of metabolite‐level effects from those exogenous chemicals in living systems is particularly important for both human health and environmental protection. However, due to the complexity of metabolic pathways, diversity of environmental chemicals, as well as the limited detection coverage of existing methods, recent metabolomics‐based research could only advance our knowledge of the biological impacts of specific pesticides, such as sulfoxaflor and triclosan,^[^ ^4^, ^5^ ^]^ by exposing cell lines or animal models to the artificial environment containing them. Hence, there is still an unmet need to monitor potential bio‐effects triggered by widely used exogenous chemicals in natural environments, especially their spatiotemporal distribution, bio‐transformations, and interactions with endogenous compounds in vivo.

Embryogenesis encompasses a series of complex events to form higher‐order organisms from a single cell and is one of the most susceptible processes to be affected by exogenous environmental chemicals.^[^ ^6^, ^7^ ^]^ Embryogenesis‐associated metabolomics analysis is significantly important for understanding how and to what extent potential environmental chemical pollutants may influence or interfere embryogenesis. However, it is particularly challenging since it requires analytical tools comprising not only single‐cell resolution but also the ability to track multiple metabolites quantitatively, simultaneously, and minimally‐destructively. So far, fluorescence spectrometry (FS), nuclear magnetic resonance spectroscopy (NMRS), and mass spectrometry (MS) are the main methods with great potential to be leveraged for single‐cell/in vivo metabolomics. Although FS enables real‐time detection,^[^ ^8^, ^9^, ^10^, ^11^ ^]^ it suffers from a number of limitations including arduous synthesis, photobleaching, and insufficient tracking channels. Besides, FS only works for known analytes, thus limiting the research scope to discover potential regulatory mechanisms underpinned by previously unknown exogenous chemicals. NMRS is a non‐destructive technique that provides quantitative information about metabolites. However, it has lower sensitivity compared to other methods, and reliable results often require relatively high sample concentrations.^[^ ^12^ ^]^ MS has emerged as a powerful alternative and a number of analytical approaches have been developed recently to couple with MS for label‐free single‐cell metabolome analyses, e.g., nanostructured surfaces,^[^ ^13^, ^14^ ^]^ post‐ionization techniques,^[^ ^15^, ^16^ ^]^ and microarrays,^[^ ^17^ ^]^ etc. However, nanostructured surface‐based MS and post‐ionization‐based MS are intrinsically sample‐destructive, so prohibit live‐cell and in vivo analysis. Microarrays generally necessitate the removal of the target cell from its primitive microenvironment followed by lysis treatment. Such post‐mortem analyses suffer from a series of limitations such as physiological perturbation and loss of contextual information. Recently, the use of micro‐sampling tools‐based capillary electrophoresis (CE) was built to combine with MS for subcellular metabolomic analysis.^[^ ^18^ ^]^ Micro‐sampling tools are highly useful in minimal‐destructive analysis, but charge‐dependent separation mechanism of CE restricts the space of metabolites that can be analyzed;^[^ ^19^ ^]^ moreover, this liquid phase extraction‐based method requires multistep processing between multiple containers, which often leads to significant sample loss, decreased detection coverage and incorrect quantitative analysis. Therefore, game‐changing single‐cell methods for embryogenesis‐associated metabolomics analysis are still urgently needed.

Cis‐diol‐containing metabolites, an important class of compounds including carbohydrates, nucleosides, nucleotides, etc., play crucial roles in many physiological processes such as signal transduction and energy storage.^[^ ^20^, ^21^, ^22^, ^23^ ^]^ Notably, endogenous cis diols are found biologically active and pivotal for biomass synthesis,^[^ ^24^ ^]^ respiratory burst,^[^ ^25^ ^]^ and signal transductions,^[^ ^26^ ^]^ during embryogenesis. Meanwhile, their spatiotemporal distribution difference during embryogenesis is associated with characteristic functions. For example, in Xenopus, adenosine triphosphate/adenosine diphosphate (ATP/ADP) ratio usually increases in pluripotent cells because of biosynthetic demands of energy,^[^ ^24^ ^]^ while it may decrease at developmental stage 12.5 because ADP is required to regulate the expression of the eye field transcription factor gene network for eye formation in embryos.^[^ ^27^ ^]^ In addition, aberrant endogenous cis‐diol metabolism during embryogenesis is also reported to be closely coupled with developmental disorders.^[^ ^28^ ^]^ For instance, dysregulated nucleosides could influence gene expression by modifying the epigenome, which results in serious genetic variation and development suspension.^[^ ^29^ ^]^ Recently, some exogenous cis‐diols have been revealed that are toxic and would interrupt endogenous cis‐diol‐associated metabolism.^[^ ^30^, ^31^ ^]^ However, in‐depth profiling of embryogenesis‐associated endogenous and exogenous cis‐diol metabolic changes remains uncovered.

**Figure 1:**
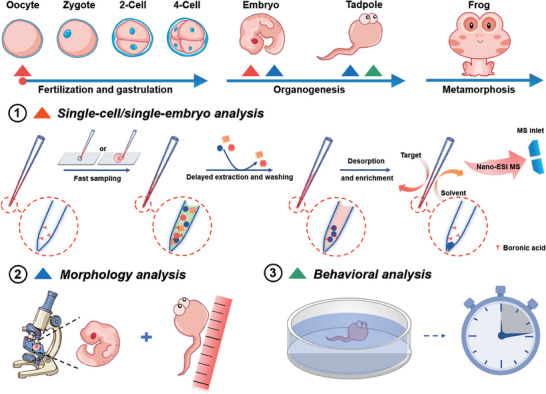
Schematic illustration of the workflow for single oocyte cells and tailbud‐stage embryo analysis. The multiscale analysis on embryogenesis begins with 1) sampling a single oocyte or single embryo and performing extraction, washing, desorption, and nano‐ESI MS‐based fingerprinting. 2) Morphology analysis for detecting eye size at Nieuwkoop‐Faber stage ≈27 and the body length of tadpoles at Nieuwkoop‐Faber stage ≈45. 3) Behavioral analysis for detecting the residence times within 1 min of each tadpole over the white and black backgrounds.

Herein, we sought to develop a solid‐phase extraction‐based micro‐biopsy platform that enables quantitative, in‐depth, and minimal‐destructive characterization of endogenous and exogenous cis‐diol metabolites during development from cellular level (single oocytes) to tissue level (different parts of tailbud‐stage embryos). This integrated platform is based on a machine learning‐empowered boronate affinity extraction‐solvent evaporation‐assisted enrichment‐mass spectrometry (BESE‐MS), which we have recently developed for sensitive serum cis‐diol fingerprinting.^[^ ^32^ ^]^ BESE‐MS is an ambient MS approach known as direct infusion mass spectrometry,^[^ ^33^, ^34^ ^]^ which offers many appealing features for targeted metabolomics, such as high specificity, high coverage, and great desalting efficiency. However, it has not reached the single‐cell resolution for minimal‐destructive analysis of living organisms. In this study, we proposed a “lab‐in‐a‐pipette” strategy via integration of all the pretreatment processes in a pipette by solid‐phase extraction technology for efficient single‐cell analysis. When compared to traditional liquid‐phase extraction‐based MS, dilution effect and sample loss were highly reduced, and sensitivity was correspondingly increased (see Figure S1, Supporting Information). In addition, two key developmental stages, oocytes and tailbud‐stage embryos of Xenopus laevis, were selected as “proof of concept” objects to investigate development‐associated cis‐diol changes (**Figure** **1**). The customized micropipette was functionalized firstly with boronic acid on the inner wall for efficiently extracting cis‐diols. Boronic acids could form five or six‐membered cyclic esters with cis‐diols under physiological environment of frog and the cyclic esters will dissociate when the environmental medium is changed to acidic conditions.^[^ ^35^, ^36^ ^]^ This pH‐controlled capture and release property made our micropipette a promising sampling tool for the extraction and enrichment of cis‐diols. The whole sampling process includes fast sampling (15 s), delayed extraction (10 min), sufficient wash steps to remove untargeted analytes, and solvent‐assisted desorption for enrichment. Fast sampling and delayed extraction endow our method with several advantages: 1) short sampling time ensures minimal‐destructive capability for metabolomic analysis; 2) fast sampling speed permits analyzing targets with fast dynamics, especially metabolites; 3) delayed extraction allows sufficient extraction adequate number of cis diols onto the boronic acid‐functionalized sampling micropipette. What's more, to demonstrate the non‐invasive nature of this method, several morphologies, and behavioral biology, including eye size and length measurement and vision test, were adopted, making this method a powerful tool for developmental biology. In this work, we first demonstrate a comprehensive cis‐diol metabolic landscape with their spatial distributions in embryos and temporal variation during embryogenesis, our results showed three extraordinary findings that were otherwise impossible or difficult to achieve. First, there are characteristically different cis‐diol metabolic signatures among oocytes, anterior and posterior parts of tailbud‐stage embryos (termed as multiscale heterogeneity). Second, halogenated cis‐diols were found heavily accumulated at the posterior part of tailbud‐stage embryos. Third, dimethachlon, a kind of exogenous fungicide that has been widely used in plants, maybe bio‐transformed and accumulate in vertebrate animals in the environment. Our results enable a deep understanding of targeted metabolic reprogramming at multiscale levels and are crucial for screening metabolite‐level effects of environmental pollutants.

## 2 Results and Discussion

### 2.1 Development of the Micro‐Biopsy‐Based Profiling Platform for Oocytes and Embryos

**Figure 2:**
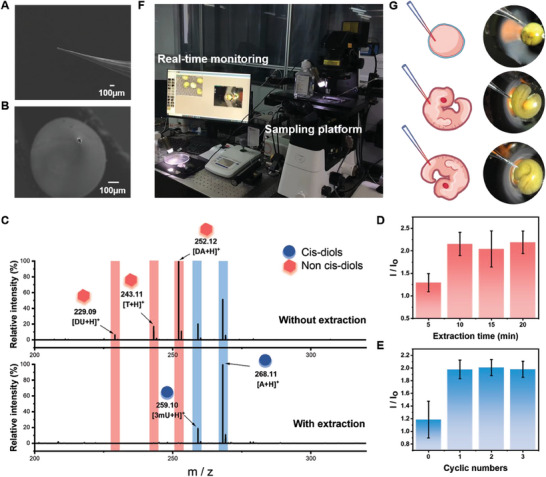
Development of a micro‐biopsy‐based profiling platform for oocytes and embryos. A, B) SEM characterization of sampling micropipettes. C) MS spectrum of direct analysis of a mixture containing an equal concentration of standard adenosine, 3‐methyluridine, 2′‐deoxyadenosine, 2′‐deoxyuridine, and thymidine and MS spectrum of compounds extracted from boronate affinity micropipette from the above solution. D) Optimization of extraction time (n = 3 independent repeats; mean ± s.d.). E) Optimization of cyclic number for the solvent evaporation step (n = 3 independent repeats; mean ± s.d.). F) Home‐built sampling platform and real‐time monitoring platform. G) Sampling process for single oocyte cells and different parts of tailbud‐stage embryos.

Boronate affinity extraction micropipettes were first fabricated for specifically sampling cis‐diols from single oocytes or single embryos. Commercial glass capillaries were laser‐pulled and then their inner‐wall were grafted with 2,4‐difluoro‐3‐formylphenylboronic (DFFPBA), which has proven to be a high‐affinity boronate affinity ligand.^[^ ^37^, ^38^, ^39^ ^]^ SEM characterization shows that the tip was not blocked after functionalization and the diameter of sampling micropipette was ≈20 µm (**Figure** **2**A, B), making it suitable for single‐cell and living embryo sampling. Because boronate affinity interaction is of paramount importance for cis‐diols extraction, we first investigated the affinity of DFFPBA‐grafted micropipettes toward cis‐diol‐containing compounds. The results show that the DFFPBA‐grafted micropipettes exhibited high affinity towards adenosine (a cis‐diol compound) while almost no affinity towards deoxyadenosine (a non‐cis‐diol compound) (Figure S2, Supporting Information), indicating successful DFFPBA modification on the inner‐wall of micropipettes and also confirming the selectivity of the micropipettes to cis‐diols. Besides, for further investigating the multi‐cis‐diols binding capability of the micropipettes, a more complex solution containing equal concentration of standard adenosine (A), 3‐methyluridine (3mU), 2′‐deoxyadenosine (DA), 2′‐deoxyuridine (DU), and thymidine (T) was tested. The MS spectra of standard solution and elution after extraction show that the boronate‐affinity probe enabled selective extraction of multiple cis‐diols (Figure 2C), which confirms the practicality of this sampling method for application to complex real samples. In addition, the detection of limited amounts of cis‐diol metabolites from the cells necessitated minimal‐destructive and ultrasensitive analysis, so the different parameters in sampling and enrichment process (fast sampling time, delayed extraction time, and cycle of solvent evaporation process) are quite important and need to be optimized. A short sampling time of 15 s was found suitable to prevent damaging cells, which is consistent with some of the reported works.^[^ ^18^ ^]^ Furthermore, by using a limited volume (1 µL) of standard solution to mimic the cells of similar size under test, it was found that delayed extraction for 10 min was appropriate and one cycle of solvent evaporation already met the detection needs (Figure 2D, E). Thus, these parameters were adopted in the following experiments. Additionally, the reproducibility test of this method is required for precise metabolomics analysis. Thus, we analyzed the signal fluctuation of extracted standard substances (adenosine and guanosine) to determine the coefficient of variation (CV). We found that CVs were lower than 13% when calibrated with internal standard (IS) (Figure S3, Supporting Information), which is well acceptable in omics analysis. As a comparison, when no IS was employed, the CV could increase to more than 20%, which pinpoints the importance of introducing IS. Further, to enable oocyte cell and in vivo sampling, a unique sampling platform was rationally designed and constructed (Figure 2F). The sampling process for single oocyte cell and tailbud‐stage embryos is shown in Figure 2G. It shows that there was no obvious content leakage after sampling, confirming the minimal‐destructive nature of this micro‐biopsy‐based method. Besides, spatial sampling in embryos was also recorded (Movies S1 and S2, Supporting Information), demonstrating the versatility of this sampling platform for single‐embryo spatial‐resolved analysis.

### 2.2 Morphology and Behavioral Biology Analysis

**Figure 3:**
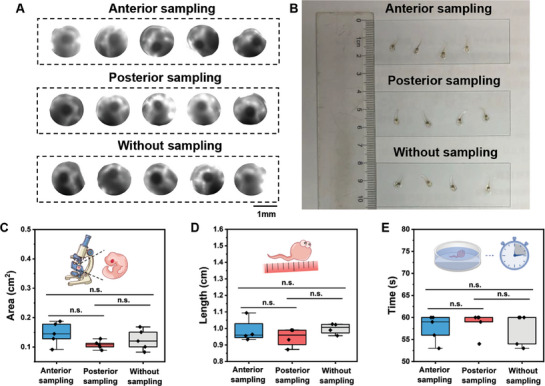
Morphology and behavioral biology analysis. A) Photos of eyes of sampled and unsampled embryos at the Nieuwkoop–Faber stage around 27 (N = 5 for each group). B) Photos of tadpoles developed from sampled and unsampled embryos at the Nieuwkoop–Faber stage around 45 (N = 4 for each group). Comparison of C) area of eyes at Nieuwkoop‐Faber stage around 27 (N = 5 for each group), D) tadpole size at Nieuwkoop‐Faber stage around 45 (N = 4 for each group), and E) time to stay at the lighter background (N = 4 for each group) between sampled and unsampled embryos (n.s. means non‐significant).

Little or even no interference with the original state of the samples is important for single‐cell and single‐embryo analysis. To verify the minimal‐destructive nature of our sampling approach to oocytes and embryos, we performed a series of experiments associated with morphology and behavioral biology to assess the damage to samples caused by our method. We compared two parameters first to explore the difference between sampled and unsampled embryos, including area of eyes at Nieuwkoop‐Faber stage ≈27 (**Figure** **3**A) and tadpole size at Nieuwkoop‐Faber stage ≈45 (Figure 3B). The results show that there was no significant difference between the unsampled and anterior prat‐sampled or posterior part‐sampled embryos in morphology (Figure 3C, D), demonstrating that at least there was no impairment to physical appearance in embryonic development via our sampling process. Because physical appearance could not be equated to behavior,^[^ ^18^ ^]^ we further evaluated the behavioral biology between sampled and unsampled embryos. It was found that all the tadpoles developed from sampled or unsampled embryos exhibited a higher preference toward lighter backgrounds, and their manners were indistinguishable depending on whether they were sampled or not (Figure 3E, Movies S3–S5, Supporting Information), indicating there was also no detectable impairment on their behavioral phenotype or even functions. In order to further assess the influence of the sampling process on embryos, we conducted an analysis of embryonic survival up until the tadpole (Nieuwkoop–Faber stage ≈45). In comparison to the control group, where all tadpoles successfully developed, we found that 92% of embryos developed into tadpoles in the anterior sampling group, and 94% of embryos developed into tadpoles in the posterior sampling group (Figure S4, Supporting Information), demonstrating that our sampling process is minimal‐destructive to the samples.

### 2.3 Mapping of Cis‐Diols in Oocytes and Tailbud‐Stage Embryos

**Figure 4:**
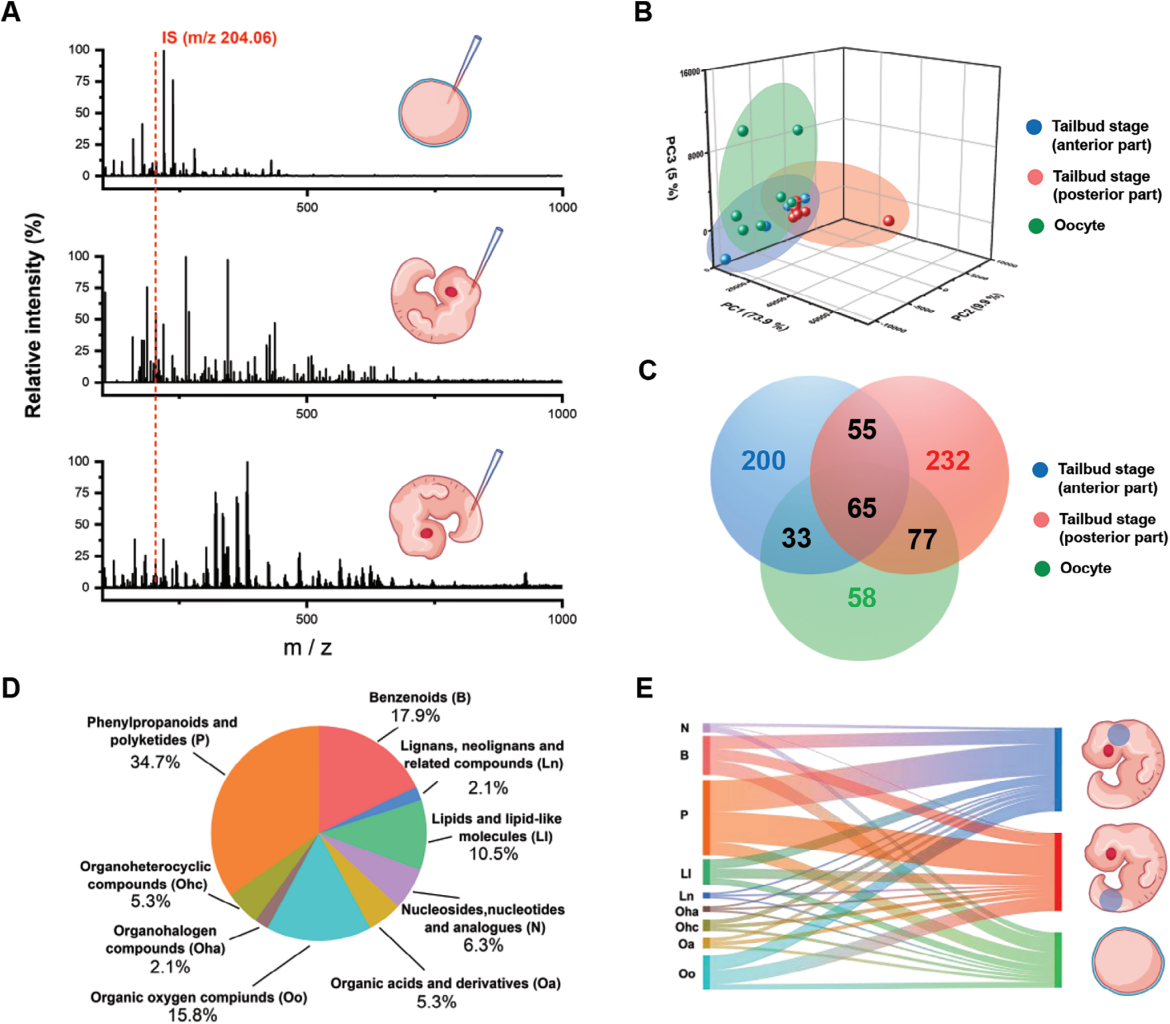
Mapping of cis‐diol metabolites of the oocyte and organogenesis‐stage embryo. Raw typical MS spectra of A) oocyte cell, anterior and posterior part of tailbud‐stage embryo. B) PCA analysis of three kinds of samples. C) Venn diagram of three kinds of samples. D) Pie chart of ClassyFire categories to classify the metabolite diversity of all annotated cis‐diols. E) Mulberry chart of annotated cis‐diols to understand the relationship of them with three kinds of samples.

Having constructed and optimized the method, we further fingerprinted cis‐diols from single oocyte cells (maternal cells before totipotent state) and tailbud‐stage embryos (a point of early organogenesis at which dozens of distinct cell types have formed encompassing the early progenitors of most major organs) with the help of nano‐ESI MS. The typical raw MS spectra of single oocytes, anterior and posterior parts of tailbud‐stage embryos are shown in **Figure** **4**A. Principal component analysis (PCA) revealed that the anterior and posterior parts of tailbud stage aggregated into a tighter cluster while oocytes tend to be scattered (Figure 4B). This indicates that the direction of cis‐diol metabolic accumulation during development is consistent. Further, the total number of detected peaks in these samples, confirmed by signal‐to‐noise ratio (S/N) >3 and must be detected in at least 3 tests of same sample groups, are 233 for oocyte cells, 353 for anterior part of tailbud‐stage embryos, and 429 for posterior part of tailbud‐stage embryos (Figure 4C). The overlapping section in the Venn diagram shows there are 65 peaks common to all three samples, indicating that some metabolites may keep bioactive not only before fertilization but also during organogenesis. At the same time, the non‐overlapping sections also demonstrate there are different cis‐diol metabolic signatures exist among three samples, confirming the valuable significance in operating spatiotemporal‐resolved analysis during embryogenesis. Then, we identified 95 metabolites (Table S1, Supporting Information) and defined them into nine super classes using the ClassyFire classification system (Figure 4D), covering a wide range of biochemicals including benzenoids (17.9%), lignans, neo‐lignans, and related compounds (2.1%), lipids and lipid‐like molecules (10.5%), nucleosides, nucleotides, and analogs (6.3%), organic acids and derivatives (5.3%), organic oxygen compounds (15.8%), organohalogen compounds (2.1%), organoheterocyclic compounds (5.3%), and phenylpropanoids and polyketides (34.7%). Additionally, the attribution of each metabolite in the three types of samples is also illustrated (Figure 4E). The results show that the benzenoids, phenylpropanoids, and polyketides dominated the metabolites in oocyte cells and tailbud‐stage embryos, respectively, indicating the difference in molecular composition in early and late stages of developing landscape.

### 2.4 Spatiotemporal Cis‐Diol Metabolome Atlas from Developing Embryo

**Figure 5:**
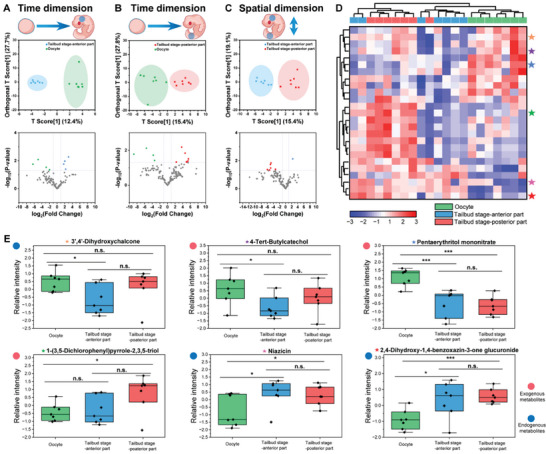
Spatiotemporal cis‐diol metabolome atlas from developing embryos. Difference in time dimension: comparison of cis‐diols between single oocyte cells and anterior A) or posterior B) part of tailbud‐stage embryos with OPLS‐DA and Volcano plot. C) Difference in spatial dimension: comparison of cis‐diols between anterior and posterior part of tailbud‐stage embryos with OPLS‐DA and Volcano plot. D) False‐color heatmap and hierarchical clustering of small molecules detected in oocytes, anterior and posterior part of tailbud‐stage embryos (N = 7 for each group). Metabolite abundances are normalized and transformed by log10 and shown in false color for the 25 statistically most significant features. E) Multiscale heterogeneity of 3′,4′‐dihydroxychalcone, 4‐tert‐butylcatechol, pentaerythritol mononitrate, 1‐(3,5‐dichlorophenyl)pyrrole‐2,3,5‐triol, niazicin and 2,4‐dihydroxy‐1,4‐benzoxazin‐3‐one glucuronide among oocytes, anterior and posterior part of tailbud‐stage embryos.

Impressive metabolic changes have been implicated during the early stage of embryogenesis.^[^ ^25^ ^]^ However, a deeper understanding of this process underpinned by cis‐diol metabolism is limited. Therefore, we next sought to comprehensively delineate the distinct spatiotemporal dynamics of embryogenesis‐associated cis‐diols. Initially, the cis‐diol metabolic changes in time dimension were investigated. Although minor group differentiation between oocytes and tailbud‐stage embryos was observed in unsupervised PCA score plots (Figure S5, Supporting Information), a clear group clustering was obtained in supervised OPLS‐DA (Orthogonal Partial Least Squares‐Discriminant Analysis) (**Figure** **5**A, B), indicating that there are great cis‐diol metabolic changes when developing from oocytes to tailbud‐stage embryos. Besides, the top fifteen features with the highest variable importance on projection (VIP) from OPLS‐DA, which have great potential to be key players in understanding temporal heterogeneity, are summarized in Figure S6 (Supporting Information). The volcano plot was also used to find the difference between the oocytes and tailbud‐stage embryos. It was found that there were more up‐regulated cis‐diols in posterior part of tailbud‐stage embryos (Figure 5B), which indicates that the cis‐diol‐associated metabolism could be more intense in the posterior part of tailbud‐stage embryos. For further understanding this process, the correlation heatmap analysis was performed (Figure S7, Supporting Information), and the results show that the developmental pattern of cis‐diol metabolic pathways was different. These findings confirmed that there are separate cis‐diol metabolic developmental patterns at specific regions of embryos during embryogenesis. More importantly, it reminds us of the importance of comparison of spatial cis‐diol heterogeneity in understanding embryogenesis. Therefore, we compared the cis‐diol profiling data of the anterior and posterior parts of tailbud‐stage embryos. Because classical PCA was found to be associated with unsatisfactory performance (Figure S8, Supporting Information), we further turned to the OPLS‐DA. It offers an outstanding performance in differentiating metabolites sampled from the two parts of embryos (Figure 5C). And the top fifteen features with highest VIP value from OPLS‐DA are also summarized in Figure S9 (Supporting Information). In addition, the volcano plot shows that there were more cis‐diols upregulated in posterior part when compared with anterior part (Figure 5C). Hence, it could be found that no matter in time dimension or spatial dimension, when zygotes develop from oocytes, through fate commitment, and into the tailbud stage, the cis‐diol‐related metabolism is obviously concentrated in the posterior part of early organogenesis. This implies that there may be more functions related to energy metabolism and signal transduction at the posterior part of early organogenesis.

In order to have a clear understanding of the cis‐diol metabolome atlas within spatiotemporal dynamics, we performed multivariate analysis to compare cis‐diol changes at multiscale levels (molecular changes between oocytes and tailbud‐stage embryos). Figure 5D shows the unsupervised hierarchical cluster analysis (HCA) and heat map of oocytes, anterior and posterior parts of tailbud‐stage embryos, which were computed based on the relative abundance of 25 of the statistically most significant metabolites. In the resulting dendrogram, the samples formed three main groups and basically coincided with their original sources, demonstrating that all of the measured samples (oocytes, anterior and posterior part of tailbud‐stage embryos) had their own type‐characteristic cis‐diol metabolic activity. In addition, 3′,4′‐dihydroxychalcone, 4‐tert‐butylcatechol (exogenous metabolites), and pentaerythritol mononitrate (exogenous metabolites) were found abundant in oocytes but rare in embryos; on the contrary, 1‐(3,5‐dichlorophenyl)pyrrole‐2,3,5‐triol (exogenous metabolites), niazicin and 2,4‐dihydroxy‐1,4‐benzoxazin‐3‐one glucuronide were found abundant in embryos but rare in oocytes (Figure 5E). The results observed above demonstrate the activity of cis diols was independent of its developmental state (oocyte or tailbud‐stage embryo) or source (endogenous or exogenous metabolites). All these findings presented herein, for the first time to the best of our knowledge, can establish a conclusion that the cis‐diols of developing embryos are heterogeneous over time and space, and the difference of distinct metabolomes are reproducible for multiple embryos of different parental origins (N = 7).

### 2.5 Expression Landscape of Halogenated Cis‐Diols and Discovery of Exogenous Pesticide

**Figure 6:**
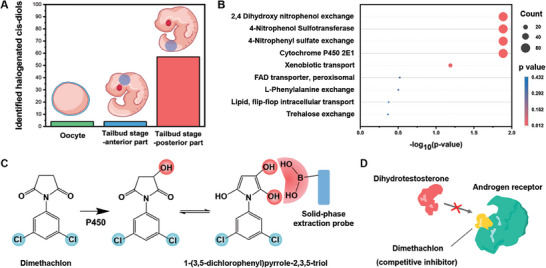
Expression Landscape of halogenated cis‐diols and discovery of exogenous pesticide. A) Comparison of halogenated cis‐diols in three kinds of samples. B) Enzyme enrichment analysis of identified cis diols. C) Illustration of how dimethachlon was converted into 1‐(3,5‐dichlorophenyl)pyrrole‐2,3,5‐triol and extracted by boronate affinity probes. D) Illustration of interaction between dimethachlon and androgen receptor.

Further investigation of the cis‐diol metabolome atlas has revealed that halogenated cis‐diol metabolites tend to accumulate preferentially in tailbud‐stage embryos. Although it has been reported that numerous marine organisms tend to sequester and incorporate halogens in their metabolism, resulting in 15–20% of newly discovered marine natural products are organohalogens,^[^ ^40^, ^41^, ^42^ ^]^ such different spatial distributions in Xenopus laevis have not been reported yet before. By using the bioinformatics tool MeHaloCoA,^[^ ^43^ ^]^ the expression difference of halogenated cis‐diols was calculated and compared. As shown in **Figure** **6**A and Tables S2–S4 (Supporting Information), it could be found that a variety of halogenated cis‐diols accumulated in the posterior part of tailbud‐stage embryos. Due to the high reactivity nature of halogens, it is reasonable to assume that bioactive reaction within cis‐diols metabolism increased at posterior part in tailbud‐stage embryos. Besides, these findings might be of great interest to natural product chemists who look for new bioactive compounds and chemical biologists who can leverage these compounds in vivo labeling. Furthermore, to explore how they were transformed in vivo, enzyme enrichment analysis was adopted (Figure 6B), and it showed the accumulation of cis diols in vertebrates could be attributed to 2,4 dihydroxy nitrophenol exchange, 4‐nitrophenol sulfotransferase, 4‐nitrophenyl sulfate exchange, and cytochrome P450 2E1. By comparing and analyzing the above enzyme enrichment analysis results with reported papers,^[^ ^44^, ^45^ ^]^ we found 1‐(3,5‐dichlorophenyl)pyrrole‐2,3,5‐triol could be converted from dimethachlon, a pesticide used for water and plant sterilization, by P450 in vivo (Figure 6C). To the best of our knowledge, although it has been revealed that dimethachlon is toxic to rats because it may lead to kidney tubular necrosis and polyuria,^[^ ^46^ ^]^ how it influences metabolic pathways of early embryo development has not been reported. From our results, it could be assumed that the hydroxylation of dimethachlon by P450 starts after fertilization and it shows a posterior‐preferred accumulation in tailbud‐stage embryos. In addition, as illustrated in Figure 6D, it has been reported recently that it could act as a competitive inhibitor to block interaction between dihydrotestosterone and androgen receptors,^[^ ^47^ ^]^ thus we may put a warning of cautious use of dimethachlon and call for more efforts towards understanding its potential environmental impact. In addition, it also reminds us that our method can be a useful tool to investigate how exogenous environmental halogenated pollutants interact and influence embryogenesis.

## 3 Conclusion

Single‐cell metabolomics has the potential to offer new insights by shedding light on the endogenous and exogenous metabolic reprogramming that accompanies embryogenesis, especially in revealing potential bio‐effects that are caused by environmental chemicals. However, the understanding of embryogenesis underpinned by specific metabolites has much lagged for the lack of proper toolsets for selective molecular fingerprinting from single cells to developing embryos. Taking cis‐diol metabolic compounds as an example, we herein presented a solid‐phase extraction‐based micro‐biopsy strategy to spatiotemporal‐resolved mapping targeted metabolomics during embryogenesis at both the cellular and tissue levels. The in‐depth characterization of endogenous and exogenous cis‐diols landscape from single oocyte cells to organogenesis‐stage embryos revealed different cis‐diol metabolites‐associated developmental patterns over time and space. Meanwhile, halogenated cis‐diols were found to have an obvious posterior‐preferred accumulation at tailbud‐stage embryos. What's more, a potential bio‐transformation and accumulation behavior of a widely used pesticide, dimethachlon, was detected. These results provide previously unavailable insights into the cell and tissue heterogeneity during embryogenesis, which is critical to delivering multiscale knowledge of metabolic reprogramming and crucial for screening metabolite‐level effects of environmental pollutants on the entire developing process. More critically, beyond the cis‐diol‐containing metabolites, we expect this scalable and reproducible approach can be generally extended to other significant metabolites, such as lipids or carbonyl‐containing metabolites. Using different affinity reagents, targeted metabolic analysis would be capable of providing more valuable molecular details for embryogenesis.

Furthermore, we believe the cis‐diol metabolome‐wide measurement of single cells and in vivo specific regions could generate important potential for comprehensively understanding specific biological events, such as unique signal transduction in disease conditions. Additionally, we anticipate this approach could be combined with assisted reproductive technology for understanding the correlation between metabolic dysregulation and reproductive diseases of humans.^[^ ^48^ ^]^ Moreover, by providing complementary information to already available genomic, transcriptomic, and proteomic data, we anticipate that targeted metabolic measurements by single‐cell/in vivo method will facilitate basic and translational research in developmental biology to help develop a holistic understanding of how cells acquire their differentiated state and give rise to distinct tissues.

## 4 Experimental Section

### Materials

Adenosine, thymidine, and 2,4‐difluoro‐3‐formyl‐phenylboronic acid (DFFPBA) were all purchased from Sigma‐Aldrich (St. Louis, MO, USA). Guanosine, 3‐methyluridine, and 2′‐deoxyadenosine were obtained from Liangwei Biotechnology (Nanjing, China). 2′‐Deoxyuridine was purchased from Topscience (Shanghai, China). Glacial acetic acid was purchased from Sinopharm Chemical Reagent (Shanghai, China). Aminopropyltriethoxysilane (APTES, 98%) and D‐galactose‐1‐13C were purchased from J&K Scientific (Shanghai, China). Anhydrous ethanol and ammonium bicarbonate were purchased from Nanjing Reagent Company (Nanjing, China). Methanol was purchased from Shanghai Macklin Biochemical (Shanghai, China). Cysteine was obtained from Aladdin (Shanghai, China). 4‐(2‐Hydroxyethyl)−1‐piperazineethanesulfonic acid (HEPES) buffer (50 mm, pH 7.5) was purchased from Yuanye Shengwu Biotechnology (Shanghai, China). Glass micropipette of 0.58 mm i.d. and 1.0 mm o.d. were purchased from DL Naturegene Life Sciences (Shanghai, China). Unless otherwise specified, all other reagents used were of analytical grade or higher purity. Water used in all the experiments was purified with a Milli‐Q Advantage A10 ultrapure water purification system (Millipore, Milford, MA, USA).

### Mass Spectrometer

Thermo Fisher LTQ‐Orbitrap XL was used in metabolomics. All MS characterizations were carried out in positive‐ion mode. Easy spray ion source parameters were as follows: spray voltage of 1.50 kV, capillary temperature of 80 °C, and tube lens at 110 V. FTMS analyzer parameters were as follows: resolution of 30 000, max inject time of 100 ms.

### Preparation of Glass Micropipettes

Borosilicate glass capillaries were pulled by P‐2000 pipette puller (Sutter Instrument, Novato, CA, USA) to obtain emitters for MS analysis. Parameters of P‐2000 were optimized as follows: HEAT = 500, FIL = 3, VEL = 25, DEL = 180, PUL = 200.

### Preparation of Boronic Acid‐Functionalized Extraction Micropipettes

The glass micropipettes were immersed in freshly prepared piranha solution for 2 h, then the micropipettes were washed with water three times and dried at room temperature. After that, the micropipettes were immersed in 4% APTES ethanol solution for 5 h. Next, they were washed with ethanol three times. Then the probes were immersed in a 100‐mL mixture solution containing 5 mg mL^−1^ sodium cyanoborohydride and 5 mg mL^−1^ DFFPBA for 24 h at room temperature, the micropipettes were washed with ethanol three times and dried at room temperature for further use.

### Characterization of Boronic Acid‐Functionalized Extraction Micropipettes

The micropipettes were immersed in 1 µL ammonium bicarbonate buffer (50 mm, pH 8.5) containing 1 mg mL^−1^ adenosine and 1 mg mL^−1^ deoxyadenosine, making the solution completely sucked into the micropipettes and keeping 1 h. After that, the micropipettes were placed on the Kimtech cleaning tissues gently to remove the solution. The micropipettes were immersed in 2 µL ammonium bicarbonate buffer (50 mm, pH 8.5) to wash micropipettes by sucking and removing the solution. The above wash process was repeated three times. Next, glass micropipettes were loaded with 1 µL 100 mm HAc solution and used as emitters for nESI MS analysis.

### Characterization of Coefficient of Variation

Boronic acid‐functionalized extraction micropipettes were immersed in 1 µL ammonium bicarbonate buffer (50 mm, pH 8.5) containing adenosine and guanosine 1 µg mL^−1^ each, making the solution completely sucked into the micropipettes and keeping for 10 min. Then the micropipettes were placed on the kimtech cleaning tissues gently to remove the solution. After that, the micropipettes were immersed in 2 µL ammonium bicarbonate buffer (50 mm, pH 8.5) to wash micropipettes by sucking and removing the solution. The above wash process was repeated three times. Next, glass micropipettes were loaded with 1 µL desorption solutions (CH_3_OH: H_2_O: HAC = 50:49:1 (V: V: V)) and were placed in a vacuum oven and the temperature was set to 40 °C for solvent evaporation. Subsequently, the glass micropipettes were used as emitters. A drop of desorption solvent or the same desorption solvent containing 50 ng mL^−1^ d‐galactose‐1‐13C was added to the tip of emitters and about 20 nL desorption solvent was sucked into the emitter tip for further nESI MS analysis.

Development of fast sampling and delayed boronate affinity extraction‐solvent evaporation assisted enrichment‐mass spectrometry: For the study of cis‐diol metabolic changes between single cells and single embryos, the boronate affinity extraction‐solvent evaporation enrichment‐mass spectrometry (BESE‐MS) which was developed recently was leveraged.^[^ ^31^ ^]^ While considering the difference in boronate affinity substrate and the difference in sample volume, there were two steps that should be re‐optimized: 1) optimization of the extraction time, and 2) optimization of the solvent evaporation cyclic numbers for desorption.

1. Optimization of extraction time: Boronic acid‐functionalized extraction micropipettes were immersed in 1 µL ammonium bicarbonate buffer (50 mM, pH 8.5) containing 1 µg mL^−1^ galactose, making the solution completely sucked into the micropipettes and keeping for 5, 10, 15, or 20 min. Then the micropipettes were placed on the Kimtech cleaning tissues gently to remove the solution. After that, the micropipettes were immersed in 2 µL ammonium bicarbonate buffer (50 mM, pH 8.5) to wash micropipettes by sucking and removing the solution. The above wash process was repeated three times. Next, glass micropipettes were loaded with 1 µL desorption solutions (CH_3_OH: H_2_O: HAC = 50:49:1, V: V) and were placed in vacuum oven and the temperature was set to 40 °C for solvent evaporation. The above desorption operation was repeated three times. Subsequently, the glass micropipettes were used as emitters. A drop of desorption solvent containing 50 ng mL^−1^ d‐galactose‐1‐13C was added to the tip of emitters and about 20 nL of desorption solvent was sucked into the emitter tip for further nESI MS analysis. A high voltage of 1.5 kV was applied to the emitter through back‐inserted copper wire. Single ion mode (SIM) within 6 m/z range was used to record the mass spectra. The measurement was repeated three times.
2. Optimization of the solvent evaporation cyclic number for desorption: Boronic acid‐functionalized extraction micropipettes were immersed in 1 µL ammonium bicarbonate buffer (50 mm, pH 8.5) containing 1 µg mL^−1^ galactose, making the solution completely sucked into the micropipettes and keeping for 10 min. After that, the procedure is almost the same as the optimization of extraction time described above except for the following difference. The desorption process in oven was operated with 0, 1, 2, or 3 times.

### Xenopus Laevis‐Related Animal Care and Embryological Experiments

All protocols concerning the humane care and handling of Xenopus laevis animals were approved by the Jiangsu Association for Laboratory Animal Science (Operator ID: 220 224 411). Adult male and female African clawed frogs were obtained from Xenopus Resource Center (Hangzhou, China) and maintained separately in breeding water boxes. Oocytes were obtained by gonadotropin‐injected female frogs, embryos were obtained from gonadotropin‐induced natural mating of two sets of parents. Obtained oocytes and embryos were dejellied in 2% cysteine solution, and cultured in 10X MMR solution (1 m NaCl, 20 mm KCl, 10 mm MgCl_2_, 20 mm CaCl_2_, 50 mm HEPES, pH 7.5) following standard protocols.^[^ ^49^ ^]^

### Micropipette Sampling and Sample Processing

Micropipette sampling was carried out on the home‐built single cell/in vivo operation platform. The sampling operation toward oocyte cells was carried out directly after they were dejellied. The developmental process of embryos was different; thus, all the embryos were monitored in the Petri dish and the embryos at tailbud stage were selected for further sampling (Figure S10, Supporting Information). Due to the high viscosity nature of the embryo contents, it was difficult to achieve effective direct sampling without pressure applied and the tip of micropipette was easily blocked. Therefore, the sampling method adopted is described below. Boronic acid‐functionalized extraction micropipettes were preloaded 1 µL ammonium bicarbonate buffer (50 mm, pH 8.5). Next, they were inserted into the oocytes or embryos under control by a three‐axis manual micromanipulator for 15 s (sampling of the anterior or posterior part of embryos was monitored by the handheld camera (Dino‐Lite, Anpeng Electronic Technology, Shenzhen, China)). After that, they were taken out of the oocytes or embryos and incubated in each centrifugal tube for 10 min to ensure effective extraction of cis‐diols. Then the micropipettes were placed on the Kimtech cleaning tissues gently to remove the solution. After that, the micropipettes were immersed in 2 µL ammonium bicarbonate buffer (50 mm, pH 8.5) to wash micropipettes by sucking and removing solution. The above wash process was repeated three times. Next, micropipettes were loaded with 1 µL desorption solutions (CH_3_OH: H_2_O: HAC = 50:49:1 (V: V)) and were placed in vacuum oven and temperature was set as 40 °C for solvent evaporation. Subsequently, micropipettes were used as emitters. A drop of desorption solvent containing 50 ng mL^−1^ d‐galactose‐1‐13C was added to the tip of emitters and about 20 nL of desorption solvent was sucked into the emitter tip for further nESI MS analysis.

### Evaluation of the Effect of Sampling on Embryonic Development

The differences between sampled and unsampled embryos were studied through three control experiments: 1) comparison of eye size around stage 27; 2) Comparison of body length after developing into tadpoles at stage around 45; 3) Comparison of the behavior after developing into tadpoles. Among them, (1) and (2) were adopted to evaluate the comprehensive impact of sampling on the development process, so two different developmental stages and evaluation parameters were selected. In terms of tadpoles with eyes it does not mean that the functions of the eyes were not damaged, so (3) was adopted to evaluate the visual function was not impaired by the sampling process.

1. Comparison of eye size around stage 27: Embryos that had developed to about stage 27 were placed under the microscope, and the exposure rate was set as 100% to locate the eyes. Then each embryo was taken pictures for further calculating the size of their eyes.
2. Comparison of body length after developing into tadpoles: Tadpoles were arranged on a slide by class and directly taken pictures for further calculating the overall body length.
3. Comparison of the behavior after developing into tadpoles: The measurement of visual function in tadpoles of the Xenopus laevis species provides a means to evaluate the effects of the sampling process. In general, when presented with a black‐and‐white background, young tadpoles at stages 44–46 tend to select a white background, while metamorphic tadpoles at stages 58–60 prefer a black background.^[^ ^50^ ^]^ This optokinetic response, which was a reflexive behavior based on vision, had been observed in various vertebrates. Taking advantage of this characteristic of tadpoles, a behavior assay was designed based on relevant literature.^[^ ^18^ ^]^ Briefly, the Petri dish was placed on a piece of black‐and‐white paper, and the edge of the outer side of the Petri dish was also pasted with black tape corresponding to the bottom. The residence times within 1 min of each tadpole over the white and black backgrounds were quantified and recorded by phone. Then the percentage of time that each tadpole spent in total over the white and black areas was analyzed by reviewing the recording.

### Statistical Analysis

The metabolite features were extracted with the help of MS‐DIAL.^[^ ^51^ ^]^ Accurate m/z values from online databases (METLIN: metlin.scripps.edu; KEGG: www.kegg.jp) were referred for metabolite peak assignments. In addition, given the difficulty in preparing pooled quality control samples to calibrate deviation, the relative error for peak assignments was set as 15 ppm. All peaks from one sample spectra were sequenced by m/z and were arranged to the matrix dataset. Then, a series of filters were applied: 1) prevalence filtering by only retaining peaks present in at least 80% of the biological samples, 2) filtering peaks with 30% missing values. After that, the dataset was subjected to MetaboAnalyst for missing values imputation (missing values will be replaced by 1/5 of min positive values of their corresponding variables), normalization (against the IS D‐galactose‐1‐13C), G‐log transformation, autoscaling, and statistical analysis (Volcano plot analysis, PCA analysis, OPLS‐DA analysis, Correlation heatmap analysis and Enzyme enrichment analysis). Furthermore, significant differences in eye size, body length, and behavior between sampled and unsampled cells (two‐sample *t*‐test, *p*‐value approach), as well as expression difference of cis‐diol metabolites between oocytes and embryos (two‐sample *t*‐test, *p*‐value approach) were performed in Origin 2023b (OriginLab Corp., Northampton, MA).

## Conflict of Interest

The authors declare no conflict of interest.

## Supporting information

Supporting Information

Supplemental Movie 1

Supplemental Movie 2

Supplemental Movie 3

Supplemental Movie 4

Supplemental Movie 5

## Data Availability

The data that support the findings of this study are available from the corresponding author upon reasonable request.
