# Supplementary material for: Chemo‐Selective Single‐Cell Metabolomics Reveals the Spatiotemporal Behavior of Exogenous Pollutants During Xenopus Laevis Embryogenesis
Source: Adv Sci (Weinh). 2023 Dec 20;11(9):2305401. doi: 10.1002/advs.202305401 (PMC10916618; doi:10.1002/advs.202305401)
Supplement: Supplementary file 1 — Supporting Information [file ADVS-11-2305401-s006.pdf]

ADVANCED SCIENCE
Open Access

Supporting Information

for *Adv. Sci.*, DOI 10.1002/advs.202305401

Chemo-Selective Single-Cell Metabolomics Reveals the Spatiotemporal Behavior of Exogenous Pollutants During Xenopus Laevis Embryogenesis

*Pengfei Li, Song Gao, Wanting Qu, Ying Li and Zhen Liu**

Supporting Information

Chemo-selective single-cell metabolomics reveals the spatiotemporal behavior of exogenous pollutants during Xenopus laevis embryogenesis

*Pengfei Li, Song Gao, Wanting Qu, Ying Li and Zhen Liu**

This PDF file includes:

Figures S1 to S10
Tables S1 to S4
Author Contributions

Other supporting materials for this manuscript include the following:

Movies S1 to S5

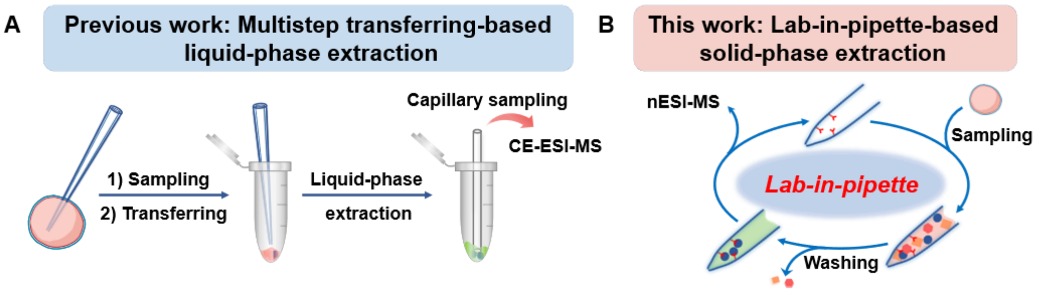

Figure S1. Comparison of the micro-sampling tools-based MS between reported papers and this work. (A) In previous work, operation process of liquid-phase extraction-based single-cell metabolomics. (B) In the present work, a solid-phase extraction-based single-cell metabolomics was developed.

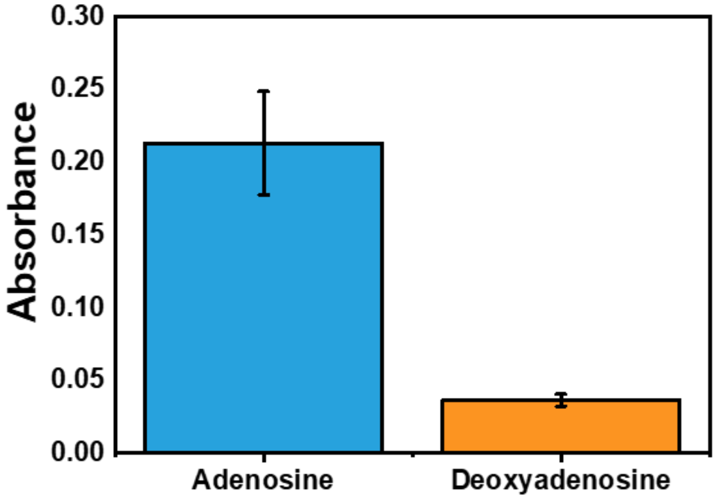

Figure S2. Comparison of the amounts of adenosine and deoxyadenosine captured by a boronic acid-functionalized extraction micropipette.

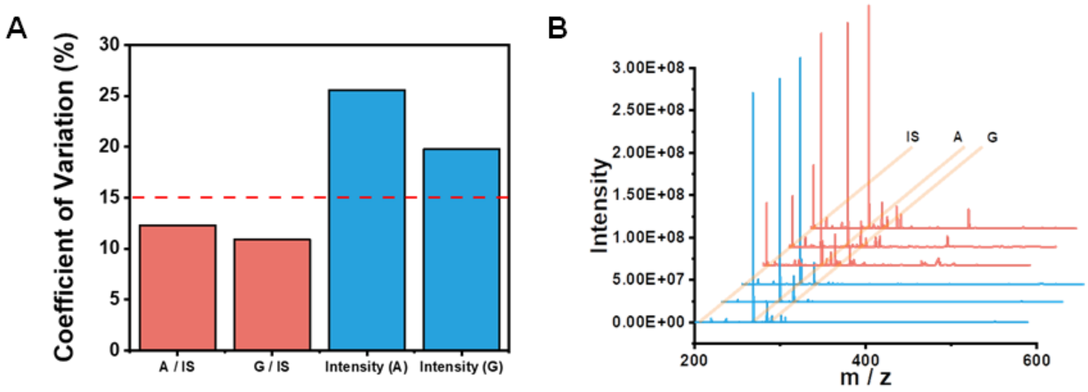

Figure S3. Assessment of reproducibility of developed method. (A) Coefficient of variation for testing adenosine and guanosine with and without of IS to calibrate. (B) Corresponding raw MS spectra for testing adenosine and guanosine.

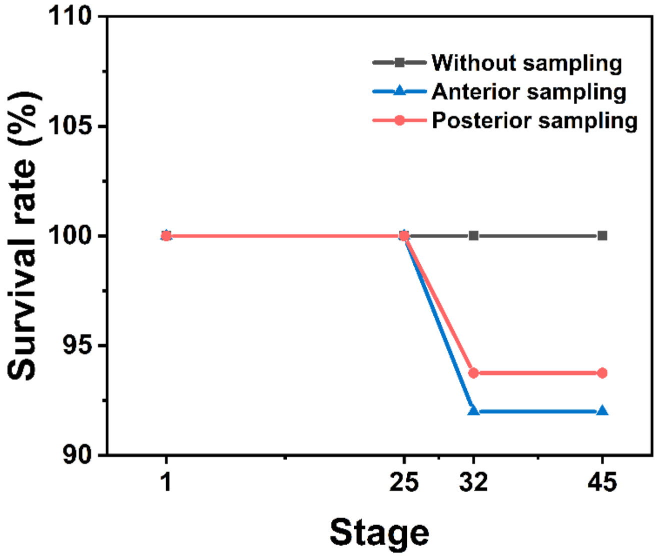

Figure S4. Survival analysis at key developmental stages (NF, Nieuwkoop-Faber) for N=41 embryos in the without sampling group, N=16 embryos in the posterior sampling group, N=25 embryos in the anterior sampling group.

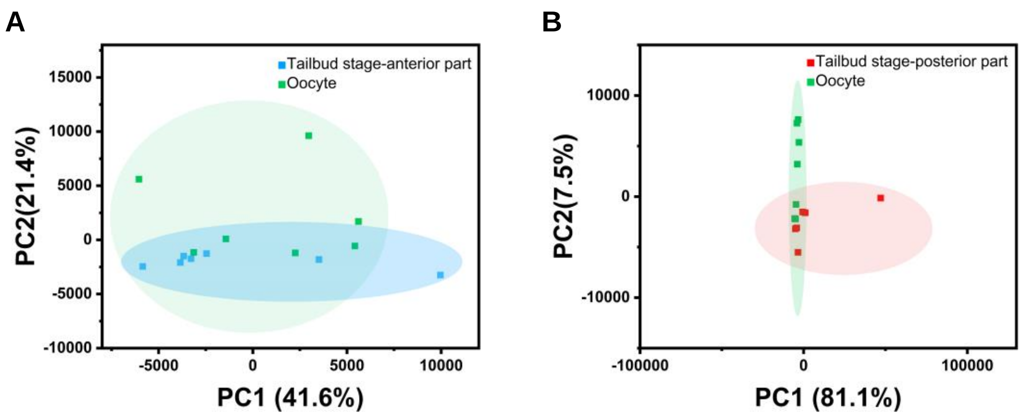

Figure S5. PCA analysis of cis-diols in time dimension. Comparison of (A) oocyte cells and anterior part of tailbud-stage embryos, and (B) oocyte cells and posterior part of tailbud-stage embryos.

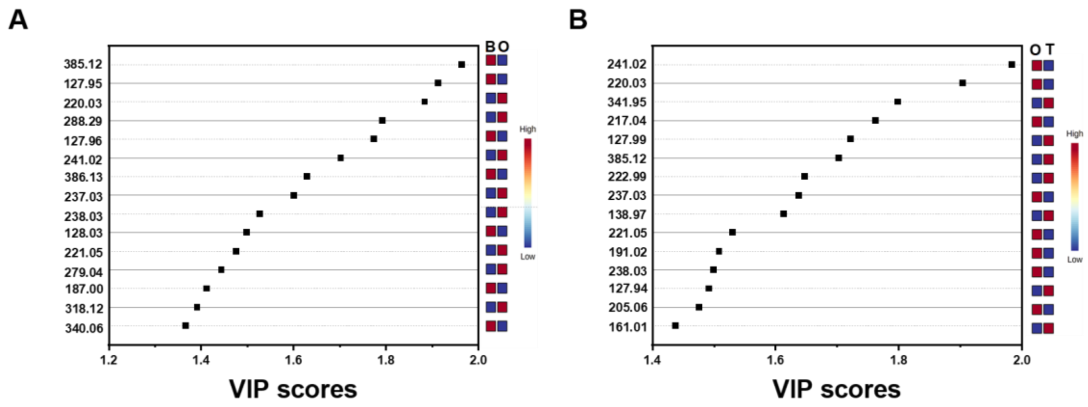

Figure S6. Significant features identified by OPLS-DA of (A) oocyte cells and anterior part of tailbud-stage embryos, and (B) oocyte cells and posterior part of tailbud-stage embryos. The features are ranked by variable importance on projection value.

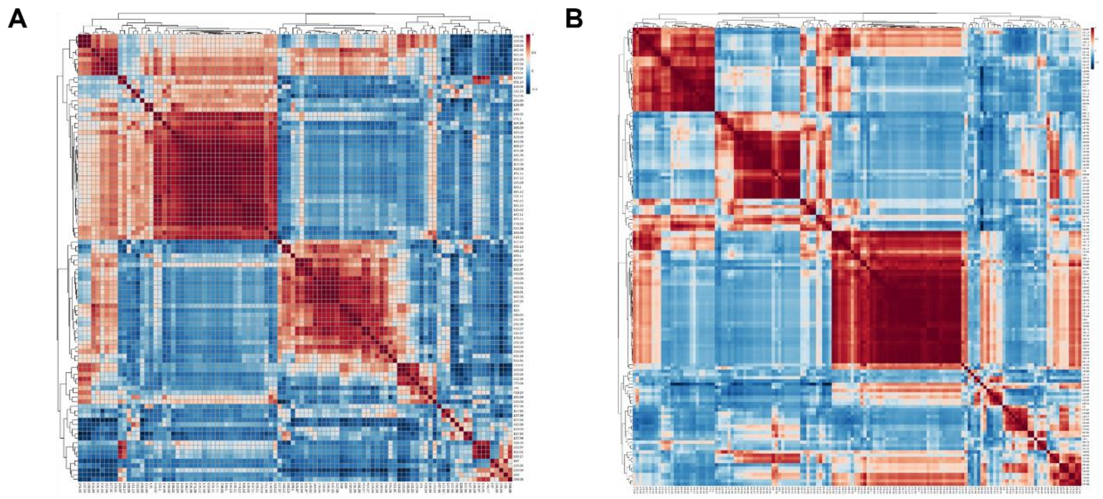

Figure S7. Correlation heatmap analysis of (A) oocyte cells and anterior part of tailbud-stage embryos, and (B) oocyte cells and posterior part of tailbud-stage embryos.

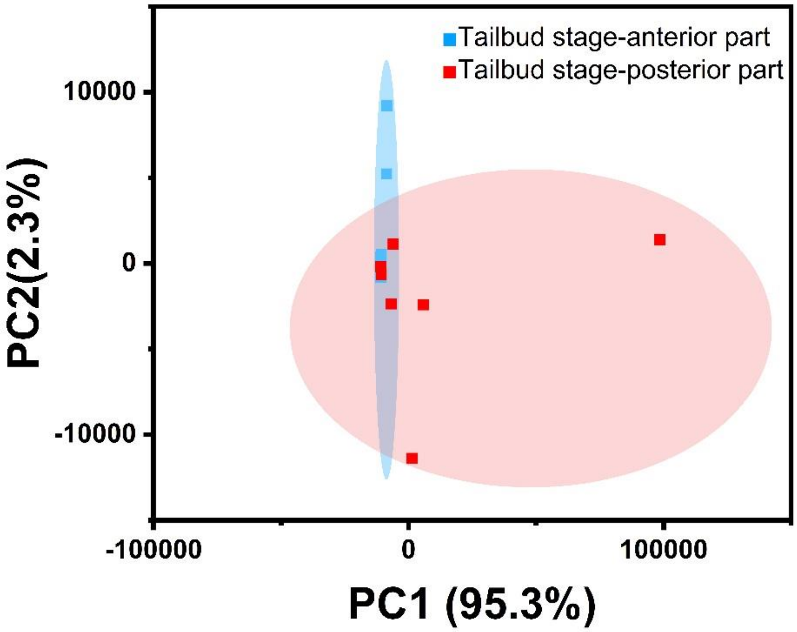

Figure S8. PCA analysis of cis-diols in spatial dimension. Comparison of anterior and posterior part of tailbud-stage embryos.

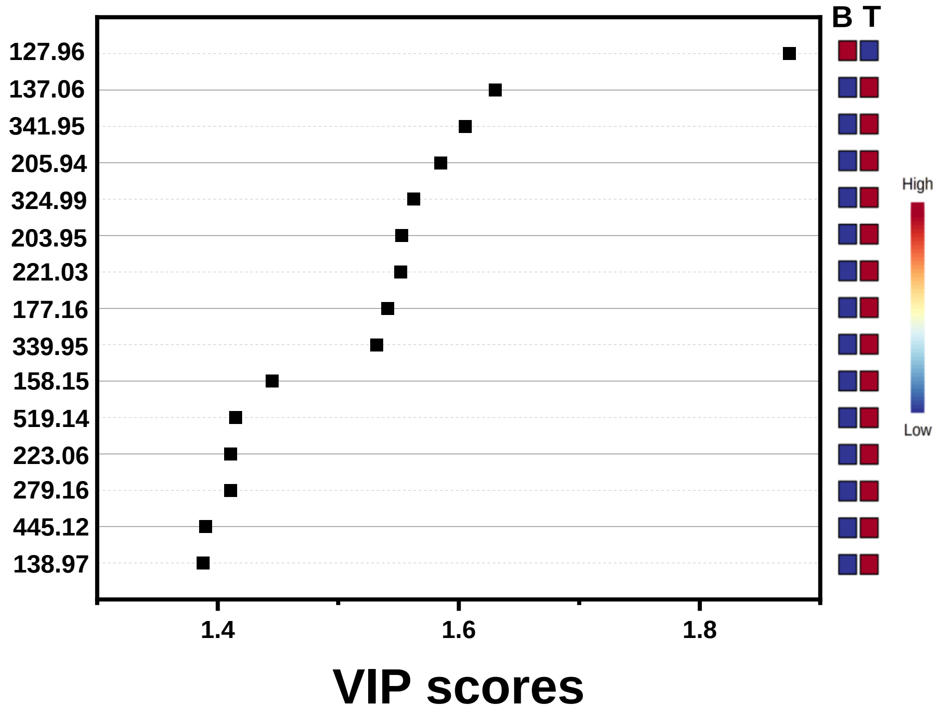

Figure S9. Significant features identified by OPLS-DA of anterior and posterior part of tailbud-stage embryos.

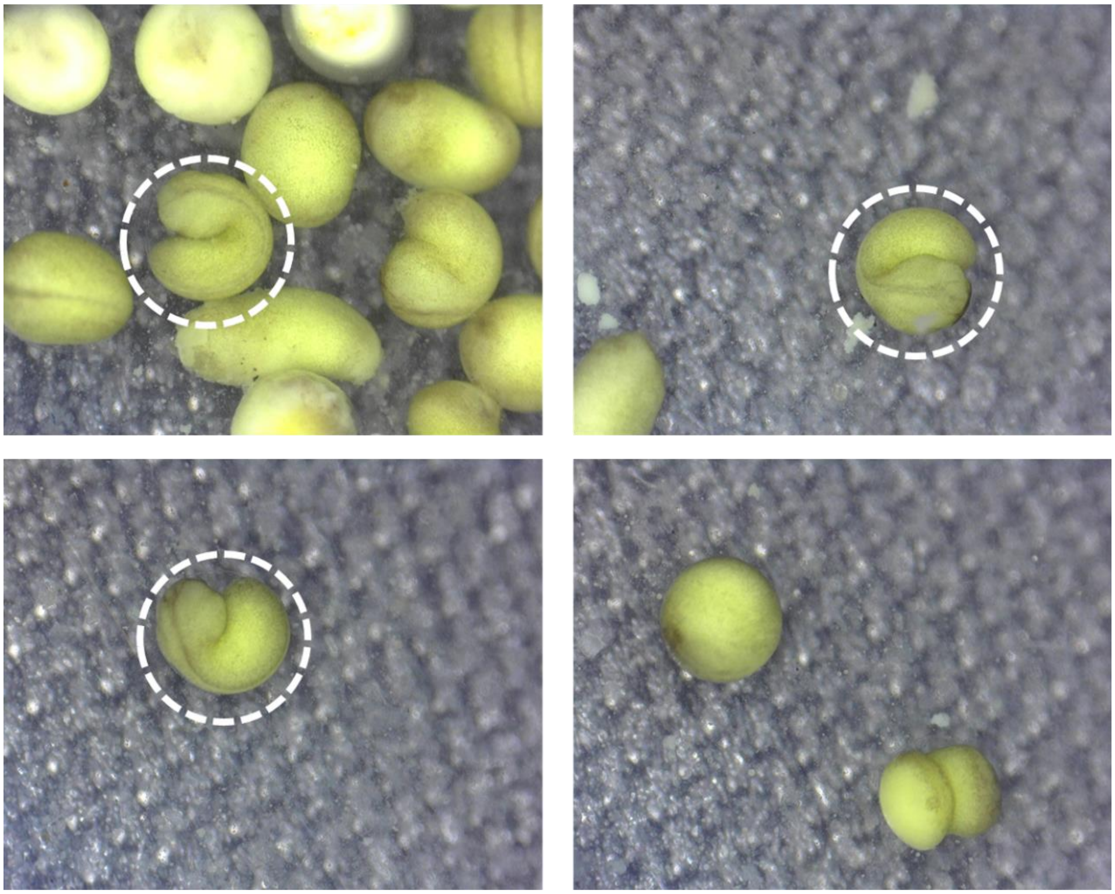

Figure S10. Developmental stage of different embryos. Tailbud-stage embryos are marked with white circle.

Table S1. Detailed information of annotated cis-diols

Name	Formula	Measured m / z	Ion type	Theoretical m / z	Relative error (ppm)
**2,3-Dihydroxypropanamide**	$C_3H_7NO_3$	128.03	M+Na	128.0318	14
**Hexanediol**<br><br>**2-Methyl-1,3-pentanediol**<br><br>**4-Methyl-1,2-dihydroxypentane**	$C_6H_{14}O_2$	141.09	M+Na	141.0886	10
**1,2,3-Trihydroxybenzene**	$C_6H_6O_3$	149.02	M+Na	149.0209	6
**serol**	$C_3H_7NO_4$	160.00	M+K	160.0007	4
**1,4-dichlorobenzene dihydrodiol**	$C_6H_6Cl_2O_2$	162.97<br>180.98	M+H-H_2O<br>M+H	162.9718<br>180.9818	11<br>10
**3-Nitrobenzene-1,2-diol**<br><br>**2,4-Dihydroxy-nitrophenol**<br><br>**4-Nitrocatechol**	$C_6H_5NO_4$	178.01	M+Na	178.0111	6
**(S)-3-(2-Methylphenoxy)propane-1,2-diol**<br><br>**(±)-threo-Anethole glycol**	$C_{10}H_{14}O_3$	183.10	M+H	183.1016	9
**(4S,8R)-8,9-Dihydroxy-p-menth-1(6)-en-2-one**	$C_{10}H_{16}O_3$	185.12	M+H	185.1172	15
**Ribonolactone**	$C_5H_8O_5$	187.00	M+K	187.0003	2
**2-carboxy-L-threo-pentonate**	$C_6H_8O_8$	191.02	M+H-H_2O	191.0203	2
**Garcinia acid**	$C_6H_8O_8$	191.02	M+H-H_2O	191.0192	4
**Isoproterenol**	$C_{11}H_{17}NO_3$	194.12	M+H-H_2O	194.1181	10
**5,6-Dihydroxy-2-methylaminotetralin**	$C_{11}H_{15}NO_2$	194.12	M+H	194.1176	13
**(R)-1,2-dimethyl-5,6-dihydroxy-tetrahydroisoquinoline**	$C_{11}H_{15}NO_2$	194.12	M+H	194.1176	13
**Arkofix**	$C_5H_{10}N_2O_5$	201.05	M+Na	201.0482	9
**Mycarose**	$C_7H_{14}O_4$	201.05	M+K	201.0524	12
**3,4-Dihydroxyphenylacetamidine**	$C_8H_{10}N_2O_2$	205.04	M+K	205.0374	13
**3,4-Dephostatin, Ethyl-**	$C_8H_{10}N_2O_3$	201.06	M+Na	205.0584	8
**4-Tert-Butylcatechol**	$C_{10}H_{14}O_2$	205.06	M+K	205.0625	12

**cis-p-Menthane-1,7,8-triol**<br><br>**7-Methyl-3-methylene-1,6,7-octanetriol**	$C_{10}H_{20}O_3$	211.13	M+Na	211.1305	2
**2,6-Dimethyl-7-octene-2,3,6-triol**	$C_{10}H_{20}O_3$	211.13	M+Na	211.1305	2
**Pentaerythritol mononitrate**	$C_5H_{11}NO_6$	220.02	M+K	220.0218	8
**Tyrphostin A25**	$C_{10}H_6N_2O_3$	241.00	M+K	241.001	4
**Chlorphenesin**	$C_9H_{11}ClO_3$	241.00	M+K	241.0028	12
**Difluorodeoxyuridine**<br><br>**1-[(4R,5R)-3,3-Difluoro-4-hydroxy-5-(hydroxymethyl)oxolan-2-yl]pyrimidine-2,4-dione**	$C_9H_{10}F_2N_2O_5$	247.05	M+H-H_2O	247.0531	12
**3',4'-Dihydroxychalcone**	$C_{15}H_{12}O_3$	279.04	M+K	279.0418	6
**Difluorodeoxycytidine**<br><br>**Gemctitabine**	$C_9H_{11}F_2N_3O_4$	286.06	M+Na	286.061	3
**Glyceryl 5-hydroxydecanoate**	$C_{13}H_{26}O_5$	301.14	M+K	301.1412	4
**2-Hydroxyenterodiol**<br><br>**4-Hydroxyenterodiol**	$C_{18}H_{22}O_5$	301.14	M+H-H_2O	301.144	13
**Ellagic acid**	$C_{14}H_6O_8$	303.01	M+H	303.0135	12
**Echinochrome A**	$C_{12}H_{10}O_7$	305.01	M+K	305.0058	14
**N2-Ethyl-2'-deoxyguanosine**<br><br>**N(6),O(2)-Dimethyladenosine**	$C_{12}H_{17}N_5O_4$	318.12	M+Na	318.1173	9
**cis-Ferulic acid 4-glucuronide**<br><br>**Fraxin**<br><br>**5-Hydroxy-6-methoxycoumarin 7-glucoside**	$C_{16}H_{18}O_{10}$	371.10	M+H	371.0973	7
**Zanamivir**	$C_{12}H_{20}N_4O_7$	371.10	M+K	371.0964	10
**(1R,3S,4S,6R)-6,9-Dihydroxyfenchone 6-O-b-D-glucoside**<br><br>**Nepetaside**<br><br>**Glucosyl 6-hydroxy-2,6-dimethyl-2E,7-octadienoate**	$C_{16}H_{26}O_8$	385.12	M+K	385.1259	15

**Niazicin**	$C_{17}H_{23}NO_7S$	386.13	M+H	386.1268	8
**Isopeonidin 3-glucoside**<br><br>**Isopeonidin 3-galactoside**<br><br>**Peonidin-3-glucoside**	$C_{22}H_{23}O_{11}$	445.12	M+H-H_2O	445.1141	13
**5-Hydroxy-3-(4-hydroxyphenyl)-6-methoxy-7-[(2S,4S,5S)-3,4,5-trihydroxy-6-(hydroxymethyl)oxan-2-yl]oxychromen-4-one**<br><br>**Leptosin**<br><br>**Kaempferide 7-glucoside**<br><br>**Swertiajaponin**<br><br>**Isoswertiajaponin**<br><br>**2-Cinnamoyl-1-galloyl-beta-D-glucopyranose**<br><br>**Pratensein 3'-glucoside**	$C_{22}H_{22}O_{11}$	445.12	M+H-H_2O	445.1135	15
**2-Methylthio-adenosine-5'-diphosphate**	$C_{11}H_{17}N_5O_{10}P_2S$	511.98	M+K	511.9803	1
**Hydroxytyrosol**	$C_8H_{10}O_3$	137.06	M+H-H_2O	137.0603	2
**(+)-threo-2-Amino-3,4-dihydroxybutanoic acid**	$C_4H_9NO_4$	158.04	M+Na	158.0424	15
**Ethyl-3,4-dephostatin**	$C_8H_{10}N_2O_3$	221.03	M+K	221.0323	10
**Trimidox**	$C_7H_8N_2O_4$	223.01	M+K	223.0116	7
**5-Amino-1-[(2R,3R,4S,5R)-3,4-dihydroxy-5-(hydroxymethyl)oxolan-2-yl]oxyimidazole-4-carboxamide**<br><br>**Charine**	$C_9H_{14}N_4O_6$	297.08	M+Na	297.0806	2
**Sylpin**<br><br>**3,4-Dihydroxycinnamoyl-(Z)-2-(3,4-dihydroxyphenyl)ethenol**	$C_{17}H_{14}O_6$	297.08	M+H-H_2O	297.0763	12
**1-(3,5-Dichlorophenyl)pyrrole-2,3,5-triol**	$C_{10}H_7Cl_2NO_3$	297.94	M+K	297.9435	12

**Dopaxanthin**	$C_{18}H_{18}N_2O_8$	373.10	M+H-H_2O	373.1036	10
**Cyanidin 7-arabinoside**<br><br>**Cyanidin 3-arabinoside**<br><br>**Cyanidin 3-xyloside**	$C_{20}H_{19}O_{10}$	401.09	M+H-H_2O	401.0878	5
**Kaempferol 3-alpha-D-arabinopyranoside**<br><br>**Salvianolic acid G**<br><br>**Luteolin 7-xyloside**<br><br>**Kaempferol 7-arabinoside**<br><br>**Scutellarein 6-xyloside**	$C_{20}H_{18}O_{10}$	401.09	M+H-H_2O	401.0873	7
**Clausarinol**	$C_{24}H_{30}O_6$	437.19	M+Na	437.1935	8
**4'-O-Methyldelphinidin 3-O-beta-D-glucoside**<br><br>**Petunidin 3-galactoside**	$C_{22}H_{23}O_{12}$	461.11	M+H-H_2O	461.109	2
**Hesperetin 7-O-glucuronide**<br><br>**Hesperetin 3'-O-glucuronide**<br><br>**Isorhamnetin 3-beta-D-glucoside**	$C_{22}H_{22}O_{12}$	461.11	M+H-H_2O	461.1084	3
**Oxypaeoniflorin**<br><br>**Mycophenolic acid glucuronide**<br><br>**Mycophenolic acid O-acyl-glucuronide**<br><br>**(R)-Isobyakangelicin 3'-glucoside**<br><br>**(R)-Byakangelicin 3'-glucoside**	$C_{23}H_{28}O_{12}$	519.14	M+Na	519.1473	14
**2-Decarboxyphyllocactin**	$C_{26}H_{29}N_2O_{14}$	593.16	M+H	593.1619	3
**Marmesin rutinoside**	$C_{26}H_{34}O_{13}$	593.16	M+K	593.1631	5
**Apiumoside**	$C_{29}H_{30}O_{12}$	593.16	M+Na	593.1629	5

Table S2. Halogenated cis-diols from oocyte cells

Intensity	M[(a)]	M1[(a)]	M2[(a)]	M1-M	M2-M	M1/M	M2/M	Filter[(b)]	Br	Cl
807454.3	308.1114	309.1157	310.1139	1.004333	2.002515	17.0564	20.52049	OK	No	OK
233381.8	327.0217	328.0107	329.0044	0.988979	1.982628	10.7469	94.06786	OK	OK	OK
354186.4	367.9504	368.9488	369.9483	0.998374	1.997828	113.4066	43.97105	OK	No	OK
401670.8	368.9488	369.9483	370.9468	0.999453	1.998019	38.77292	40.1046	OK	No	OK

(a) "M", "M1" and "M2" represent the values for the m/z of the target metabolite, the m/z of its ^{13}C isotope, and the m/z of its ^{37}Cl or ^{81}Br isotope, respectively.

(b) The presence of a specific halogen atom in each metabolite can be determined by evaluating certain criteria, such as the relative intensity percentage of [M + 2]/[M] (>30% for Cl OK, and >90% for Br OK).

Table S3. Halogenated cis-diols from anterior part of tailbud-stage embryos

Intensity	M[(a)]	M1[(a)]	M2[(a)]	M1-M	M2-M	M1/M	M2/M	Filter[(b)]	Br	Cl
13467992	361.9393	362.9348	363.9372	0.995506	1.99793	6.95989	41.37976	OK	No	OK
8545251	367.9485	368.9653	369.9534	1.016807	2.004858	17.09061	96.31188	OK	OK	OK
13354312	367.9516	368.9625	369.9509	1.010849	1.999298	6.440837	41.23473	OK	No	OK
132993.4	684.278	685.2821	686.2641	1.004167	1.986101	142.3354	93.55499	OK	OK	OK

(a) "M", "M1" and "M2" represent the values for the m/z of the target metabolite, the m/z of its ^{13}C isotope, and the m/z of its ^{37}Cl or ^{81}Br isotope, respectively.

(b) The presence of a specific halogen atom in each metabolite can be determined by evaluating certain criteria, such as the relative intensity percentage of [M + 2]/[M] (>30% for Cl OK, and >90% for Br OK).

Table S4. Halogenated cis-diols from posterior part of tailbud-stage embryos

Intensity	M[(a)]	M1[(a)]	M2[(a)]	M1-M	M2-M	M1/M	M2/M	Filter[(b)]	Br	Cl
367358.8	166.0167	167.0153	168.0196	0.998655	2.002925	64.67	275.7305	OK	OK	OK
46945556	297.9421.	298.9425	299.9464	1.000424	2.004332	7.418308	43.27008	OK	No	OK
4983407	320.91	321.9242	322.9083	1.014229	1.998317	106.2019	89.06896	OK	No	OK
37900944	334.927	335.929	336.9238	1.001969	1.996808	7.612971	91.42828	OK	OK	OK
34652180	336.9238	337.9253	338.9186	1.001547	1.994833	7.110221	21.61394	OK	No	OK
6520888	339.9557	340.9425	341.951	0.986819	1.995346	9.763078	37.92355	OK	No	OK
26558358	345.9235	346.9257	347.9224	1.00221	1.998962	9.507419	22.64324	OK	No	OK
69826272	381.8241	382.8265	383.8215	1.002422	1.997434	5.946236	134.7536	OK	OK	OK
94093392	383.8215	384.8261	385.8204	1.004597	1.998836	5.175469	43.7451	OK	No	OK
4014113	400.8473	401.832	402.8464	0.984669	1.999066	57.78814	43.3486	OK	No	OK
2319681	401.832	402.8464	403.8316	1.014396	1.999618	75.01297	37.84182	OK	No	OK
1239667	431.8807	432.8852	433.8882	1.004575	2.007535	60.85163	221.8242	OK	OK	OK
2514190	454.5118	455.5126	456.5098	1.000852	1.998033	143.5202	73.16897	OK	No	OK
1422051	503.7675	504.7836	505.7636	1.016126	1.996149	51.24678	198.2217	OK	OK	OK
22022686	520.77	521.761	522.7688	0.990997	1.998817	10.73394	174.3613	OK	OK	OK
206674.8	521.0257	522.0117	523.0007	0.986022	1.974965	57.4798	139.9471	OK	OK	OK
38399044	522.7688	523.7725	524.7655	1.003727	1.996725	8.735816	68.66775	OK	No	OK
26367760	524.7655	525.7705	526.7646	1.004931	1.999053	8.869533	28.57833	OK	No	OK
16550734	562.7803	563.7852	564.7775	1.004882	1.99725	10.94308	181.1691	OK	OK	OK
29984812	564.7775	565.7827	566.7752	1.005156	1.997707	10.78624	67.08203	OK	No	OK
20114422	566.7752	567.7806	568.7733	1.005386	1.998078	9.566805	35.47075	OK	No	OK
10602748	580.791	581.8074	582.7893	1.01642	1.998311	13.15425	175.1179	OK	OK	OK
18567306	582.7893	583.7897	584.7873	1.000361	1.997958	10.51228	63.76079	OK	No	OK
11838661	584.7873	585.7907	586.7848	1.003357	1.997512	8.73178	27.99093	OK	No	OK
5087534	622.8031	623.804	624.7995	1.000893	1.996451	16.74572	173.5665	OK	OK	OK
8830253	624.7995	625.8036	626.7982	1.004051	1.998669	18.18109	69.64773	OK	No	OK
6150071	626.7982	627.8012	628.7947	1.002934	1.996515	14.28724	28.20503	OK	No	OK

1363132	644.7309	645.7025	646.7113	0.971568	1.980412	44.01024	90.12024	OK	OK	OK
1258242	659.7166	660.7043	661.7122	0.987713	1.995564	81.83614	221.0707	OK	OK	OK
2781606	661.7122	662.7029	663.7133	0.990737	2.001141	43.70969	95.57289	OK	OK	OK
2721549	665.8155	666.8081	667.8166	0.99268	2.001132	55.39084	188.4237	OK	OK	OK
9158259	701.7272	702.7324	703.7256	1.005138	1.998343	13.19678	214.0805	OK	OK	OK
19606050	703.7256	704.728	705.7254	1.002374	1.999808	13.58864	93.45918	OK	OK	OK
18323654	705.7254	706.725	707.7195	0.999555	1.994124	13.02934	44.37728	OK	No	OK
8131540	707.7195	708.7233	709.7221	1.003795	2.002583	10.7914	22.10366	OK	No	OK
3443044	721.735	722.7578	723.7312	1.022851	1.996219	31.05797	72.89684	OK	No	OK
3281148	743.7359	744.7424	745.7393	1.006509	2.003358	22.68607	208.7203	OK	OK	OK
6848421	745.7393	746.7403	747.7356	1.001001	1.996293	25.01686	96.31439	OK	OK	OK
6596015	747.7356	748.7406	749.7321	1.005019	1.996563	23.87555	42.25367	OK	No	OK
3259862	782.6476	783.6571	784.6491	1.009405	2.001407	24.21669	115.8278	OK	OK	OK
3775826	784.6491	785.6576	786.6675	1.008497	2.018481	19.59214	58.38077	OK	No	OK
2204356	786.6675	787.6757	788.6442	1.008128	1.976702	20.38171	33.70703	OK	No	OK
2959416	822.6619	823.6733	824.6583	1.01145	1.996465	17.80926	265.6281	OK	OK	OK
7861040	824.6583	825.6678	826.6567	1.009505	1.998375	23.84684	116.719	OK	OK	OK
9175327	826.6567	827.6631	828.6533	1.006409	1.996637	20.1215	63.33241	OK	No	OK
5810956	828.6533	829.6634	830.6572	1.010058	2.003879	22.61617	46.62731	OK	No	OK
7700254	842.6685	843.676	844.6676	1.007537	1.999097	14.55979	118.3988	OK	OK	OK
9117011	844.6676	845.6714	846.6653	1.003797	1.9977	12.8242	55.56467	OK	No	OK
5065838	846.6653	847.6711	848.6689	1.005884	2.003671	18.21672	37.64904	OK	No	OK
6618596	884.6837	885.6884	886.6848	1.004721	2.001182	16.93228	103.5574	OK	OK	OK
6854047	886.6848	887.683	888.6828	0.998144	1.997972	22.83223	61.36721	OK	No	OK
2562707	909.682	910.686	911.6851	1.003944	2.003102	24.15817	119.8051	OK	OK	OK
3070253	911.6851	912.6841	913.6848	0.999006	1.999655	34.80484	67.05242	OK	No	OK
2058679	913.6848	914.6766	915.6826	0.991845	1.997856	52.00958	36.52257	OK	No	OK
9046484	926.6952	927.6975	928.691	1.002373	1.995816	20.43011	112.3625	OK	OK	OK
10164857	928.691	929.6967	930.6933	1.005679	2.002269	20.27196	58.52591	OK	No	OK
5949075	930.6933	931.6921	932.6906	0.998805	1.997298	23.00991	36.10477	OK	No	OK

(a) "M", "M1" and "M2" represent the values for the m/z of the target metabolite, the m/z of its ^{13}C isotope, and the m/z of its ^{37}Cl or ^{81}Br isotope, respectively.

(b) The presence of a specific halogen atom in each metabolite can be determined by evaluating certain criteria, such as the relative intensity percentage of [M + 2]/[M] (>30% for Cl OK, and >90% for Br OK).

Author contributions

Z. L. and P. L. conceived the idea. P. L. carried out the materials synthesis, MS experiments and data analysis. S. G., W. Q. and Y. L. helped with the sampling process. Z.L. supervised the project. Z. L. and P. L. wrote the manuscript.
